# Anti-Ulcerogenic Properties of *Lycium chinense* Mill Extracts against Ethanol-Induced Acute Gastric Lesion in Animal Models and Its Active Constituents

**DOI:** 10.3390/molecules201219867

**Published:** 2015-12-16

**Authors:** Opeyemi J. Olatunji, Hongxia Chen, Yifeng Zhou

**Affiliations:** 1 Institute of Botany, Jiangsu Province and Chinese Academy of Sciences, Nanjing 210014, China; pere@fastermail.com; 2 School of Pharmacy, Jiangsu University, Zhenjiang 212013, China; chhx2011@ujs.edu.cn; 3 Dongtai Institute of Tidal Flats, Nanjing Branch of Chinese Academy of Sciences, Dongtai 224200, China

**Keywords:** *Lycium chinense* Mill, gastric ulcer, ethanol, gastroprotective

## Abstract

The objective of this study was to explore the gastroprotective properties of the aerial part of *Lycium chinense* Mill (LCA) against ethanol-induced gastric mucosa lesions in mice models. Administration of LCA at doses of 50, 100, 200 and 400 mg/kg body weight prior to ethanol consumption dose dependently inhibited gastric ulcers. The gastric mucosal injury was analyzed by gastric juice acidity, glutathione (GSH), superoxide dismutase (SOD), malondialdehyde (MDA), myeloperoxidase (MPO) activities. Furthermore, the levels of the inflammatory mediators, tumor necrosis factor-α (TNF-α), interleukin-6 (IL-6) and interleukin-1β (IL-1β) in serum were also analyzed using ELISA. Pathological changes were also observed with the aid of hematoxylin-eosin (HE) staining. Our results indicated that LCA significantly reduced the levels of MPO, MDA and increased SOD and GSH activities. Furthermore, LCA also significantly inhibited the levels of TNF-α, IL-6, and IL-1β in the serum of ulcerated mice in a dose dependent manner. Immunohistological analysis indicated that LCA also significantly attenuated the overexpression of nuclear factor-κB in pretreated mice models. This findings suggests *Lycium chinense* Mill possesses gastroprotective properties against ethanol-induced gastric injury and could be a possible therapeutic intervention in the treatment and management of gastric ulcers.

## 1. Introduction

Gastric ulcer is a gastrointestinal disorder with multiple causes that occurs mainly in the stomach and duodenum [1]. The hallmarks that characterize gastric ulcer include necrosis, reduction of blood flow, neutrophils infiltration, oxidative stress and inflammatory mediator secretions [2]. Gastric ulcers occur as a result of an imbalance between aggressive factors (acid and pepsin secretion, infection from *Helicobacter pylori*, poor diet, excessive alcohol consumption, ROS and indiscriminate use of nonsteroidal anti-inflammatory drugs) and cytoprotective factors (mucus secretion, bicarbonate, prostaglandins, mucosal blood flow, enzymatic and non-enzymatic antioxidants). These factors could possibly contribute to gastric mucosa injury [3,4]. It has also been reported that other internal factors play critical roles in the pathophysiology involved in the protection of the gastric mucosa, such factors include, somatostatin, nitric oxide (NO) and sulfhydryl compounds [5].

The treatment options available for the management of gastric ulcer tend towards pain relief, healing and prevention of its recurrence [6]. Antacids, cytoprotective agents, muscarinic antagonists, H2 receptor antagonists and proton pump inhibitors are the available drugs for the treatment of gastric ulcer [7,8]. It is believed that drugs that could effectively treat or protect against gastric ulcer should have the ability to suppress the secretion of interleukin-6 (IL-6), tumor necrosis factor-alpha (TNF-α), scavenge ROS and/or stimulate mucosal defense mechanism [9,10].

*Lycium chinense* Mill is a plant that belongs to the Solanaceae family and is found in Southern America, Southern Africa and some Asian countries, especially China. Seven of the species of the genus *Lycium* and three varieties has been identified and is widely distributed in the Northern part of China [11]. Plants in this species, especially *Lycium barbarum* and *Lycium chinense*, are famous traditional Chinese medicines (TCMs) which are recorded in the Chinese Pharmacopoeia. The root, seeds, fruits and leaves of the plants are used in TCM as treatments for diabetes, cough, pneumonia, hematemesis, night-sweats and inflammatory disorders [12]. Bioactive components such as alkaloids, flavonoids, terpenes, organic acids, and their derivatives have been reportedly isolated from various parts of the plant [13,14,15].

The unpleasant adverse effects that often accompany the use of modern synthetic medicines for the treatment of gastric ulcer has warranted a search for effective indigenous drugs from medicinal plants which are relatively safer as better alternatives for the treatment of gastric ulcer. Thus the objective of this study is to investigate and determine the antiulcer effects of *Lycium chinense* Mill (LCA) against ethanol-induced gastric ulcer and to investigate its effects on oxidative stress markers, TNF-α, NF-κBp65, IL-1β and IL-6 which participates in immune and inflammatory responses.

## 2. Results

### 2.1. Identification of Isolated Compounds

**Figure 1:**
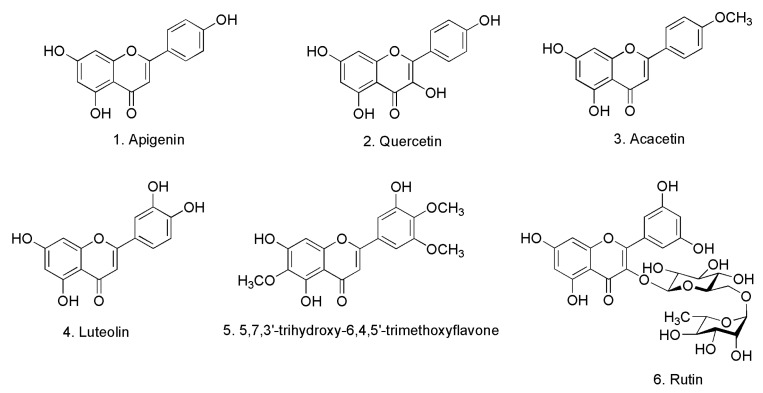
Chemical structures of the isolated compounds from LCA.

The chemical investigation of the EtOAc extracts of the aerial parts of *Lycium chinense* Mill (LCA) led to the isolation and identification of six compounds (Figure 1). These compounds were identified with the aid of complete spectroscopic analyses, including UV, IR, MS and NMR spectroscopy and by comparing their spectral data with those of previously reported in the literature. The compounds are apigenin, quercetin, acacetin, luteolin, 5,7,3′-trihydroxy-6,4,5′-trimethoxyflavone and rutin. In addition to the isolated compounds, HPLC-ESI-MS analysis of the LCA extracts indicated the presence of additional compounds identified as kaempferol, coumaric acid, ferulic acid and vanillic acid.

### 2.2. Effect of LCA on Gastric Juice Acid Content (pH)

**Figure 2:**
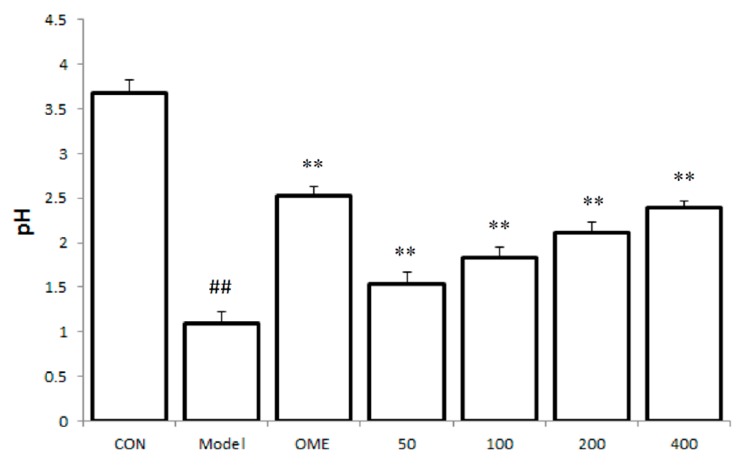
Effect of LCA on gastric juice acid content (pH). Data are expressed as the mean ± SD (*n* = 8). ##: *p* < 0.01 *vs.* control group. **: *p* < 0.01 *vs.* model group. (CON = control; OME = omeprazole).

The gastric fluid obtained from the stomach of the model group indicated a significant decrease in the pH when compared with the control mice group. However, a significant increase in the pH was observed in mice groups pretreated with LCA prior to ulcer induction when compared with the model group. The increase in the pH was observed to be dose dependently (Figure 2).

### 2.3. Effect of LCA on MPO Activity

**Figure 3:**
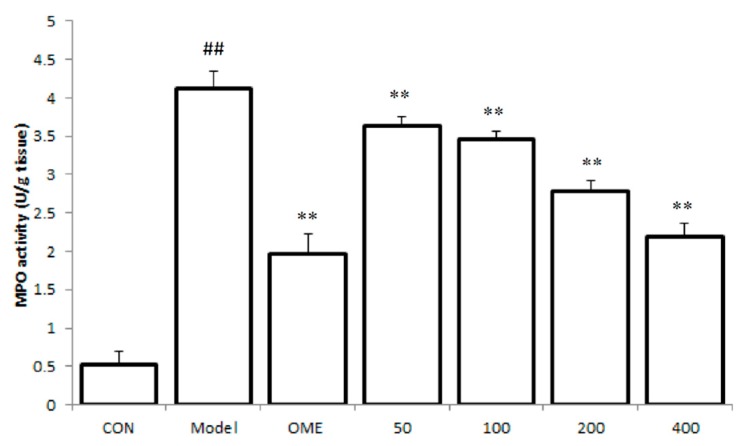
Effect of LCA on MPO activity. Data are expressed as the mean ± SD (*n* = 8). ##: *p* < 0.01 *vs.* control group. **: *p* < 0.01 *vs.* model group. (CON = control; OME = omeprazole).

The accumulation of leukocytes in the gastric mucosa after ethanol-induced lesions was assessed by evaluating gastric MPO activity. The level of MPO was observed to be significantly increased in the model mice group as compared to the control group. As indicated in Figure 3 pretreatment with LCA significantly down regulated the increased levels of MPO activity in the gastric tissues of treated mice in a dose dependent manner (*p <* 0.01).

### 2.4. Effect of LCA on MDA and SOD and GSH Levels

**Figure 4:**
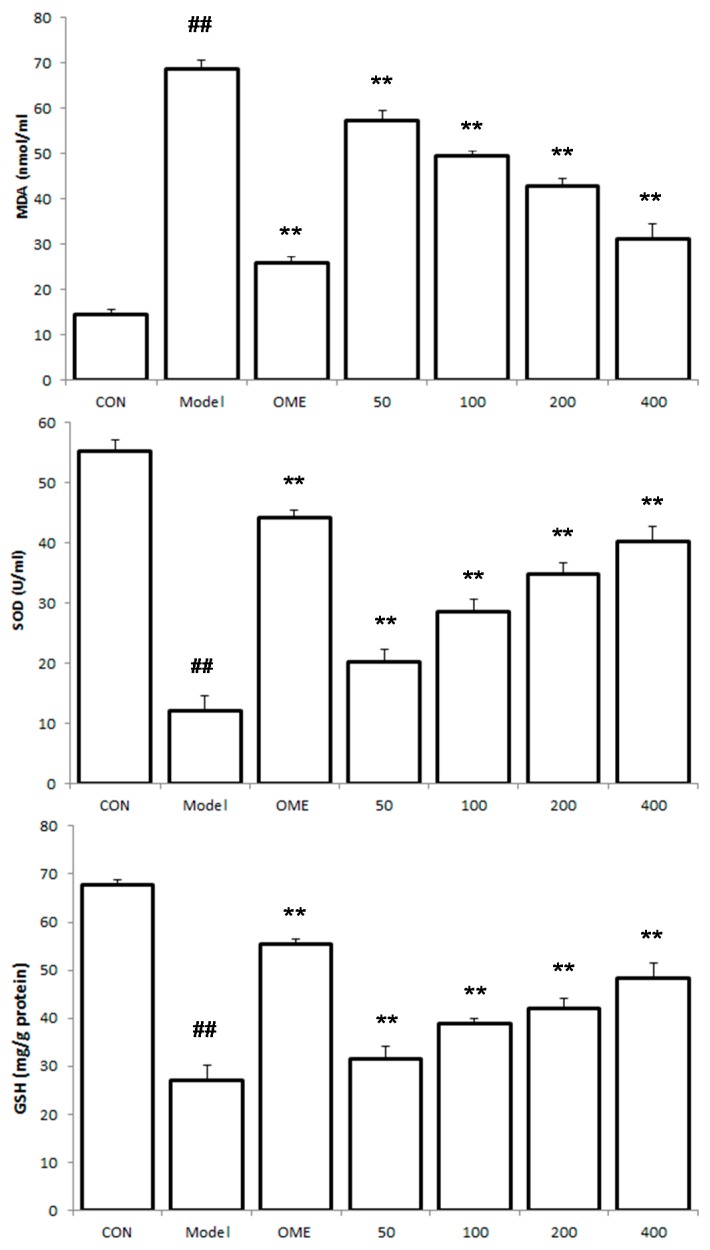
Effect of LCA on MDA, SOD and GSH levels. Data are expressed as the mean ± SD (*n* = 8). ##: *p* < 0.01 *vs.* control group. **: *p* < 0.01 *vs.* model group. (CON = control; OME = omeprazole).

MDA serum level was found to be increased, while serum levels of SOD and GSH decreased in the model mice (Figure 4). However, LCA markedly down regulated the increased levels of serum MDA and up regulated SOD and GSH levels in pretreated mice groups in a concentration dependent manner (*p <* 0.01).

### 2.5. Effect of LCA on Cytokine Levels in the Serum

**Figure 5:**
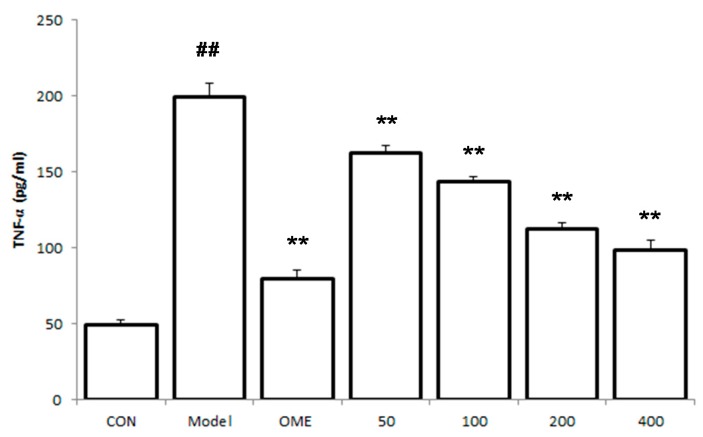

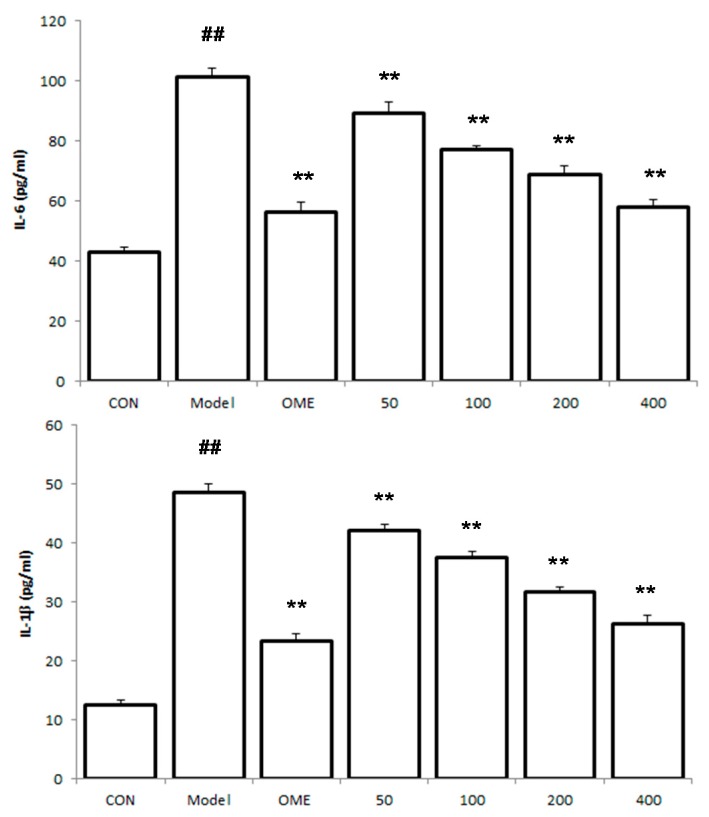
Effects of LCA on cytokine levels in the serum. Data are expressed as the mean ± SD (*n* = 8). ##: *p* < 0.01 *vs.* control group. **: *p* < 0.01 *vs.* model group. (CON = control; OME = omeprazole).

The levels of the pro-inflammatory cytokines TNF-α, IL-6 and IL-1β in the serum were observed to be significantly increased in the model mice group (Figure 5). LCA at the pretreated doses down regulated the levels of TNF-α, IL-6 and IL-1β in a concentration dependent manner.

### 2.6. Histology of Gastric Lesions

**Figure 6:**
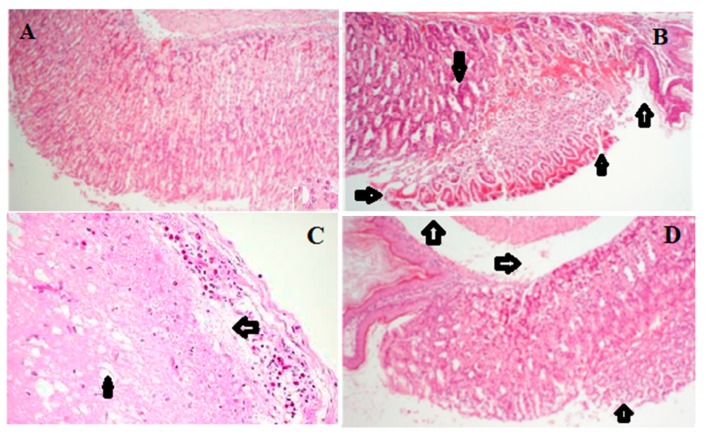

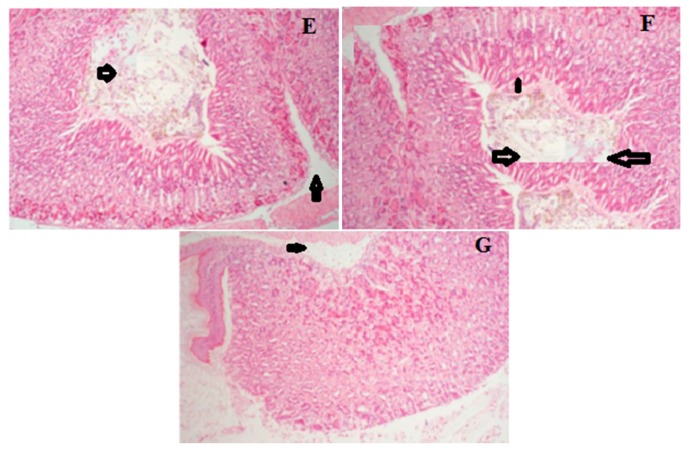
Effects of LCA on histopathological gastric lesions in mice. (**A**) Control; (**B**) model; (**C**) ethanol + OME 20 mg/kg; (**D**) ethanol + LCA 50 mg/kg; (**E**) ethanol + LCA 100 mg/kg; (**F**) ethanol + LCA 200 mg/kg; (**G**) ethanol + LCA 400 mg/kg. Arrow indicating necrotic lesions, oedema, leukocyte infiltration and disrupted cell architecture (Original magnification 100×).

Histological examination of the gastric mucosa of ulcerated mice models by H & E staining indicated gross damages to gastric mucosa inflicted by the ethanol administration. Necrotic lesions, oedema, leukocyte infiltration of the submucosal layer, as well as a disrupted cell architecture were observed in the model mice group as compared with the normal control mice. However, mice groups that received prior pretreatment with LCA showed marked protection of the gastric mucosa as indicated in the reduced ulcer area, leukocyte infiltration and submucosal edema (Figure 6; Table 1).

**Table 1:** Protective effect of LCA on ethanol induced microscopic damage in the gastric mucosa.

Groups	Doses (mg/kg)	Hemorrhagic Damage	Edema	Epithelial Cell Loss	Inflammatory Cells
Control	-	0.00 ± 0.00	0.00 ± 0.00	0.00 ± 0.00	0.00 ± 0.00
Model	-	3.78 ± 0.32 ^##^	3.83 ± 0.62 ^##^	3.62 ± 0.17 ^##^	2.54 ± 0.48 ^##^
OME	20	1.31 ± 0.44 **	1.58 ± 0.36 **	1.47 ± 0.24 **	1.30 ± 0.33 **
LCA	50	3.42 ± 0.41 **	3.65 ± 0.17 **	3.34 ± 0.27 **	2.21 ± 0.28 **
	100	2.97 ± 0.14 **	2. 87 ± 0.52 **	2.90 ± 0.38 **	1.96 ± 0.12 **
	200	2.48 ± 0.47 **	2.43 ± 0.12 **	2.62 ± 0.37 **	1.81 ± 0.23 **
	400	2.21 ± 0.29 **	1.82 ± 0.56 **	2.22 ± 0.35 **	1.50 ± 0.10 **

The values are expressed as mean ± SEM and analyzed by ANOVA followed by Tukey test. ^##^: *p* < 0.01 *vs.* control group. **: *p* < 0.01 *vs.* model group. Epithelial cell loss (score: 0–3), edema in the upper mucosa (score: 0–4), hemorrhagic damage (score: 0–4), and the presence of inflammatory cells (score: 0–3), yielding a maximum total score of 14.

### 2.7. Effect of LCA on NF-κBp65 Expression

**Figure 7:**
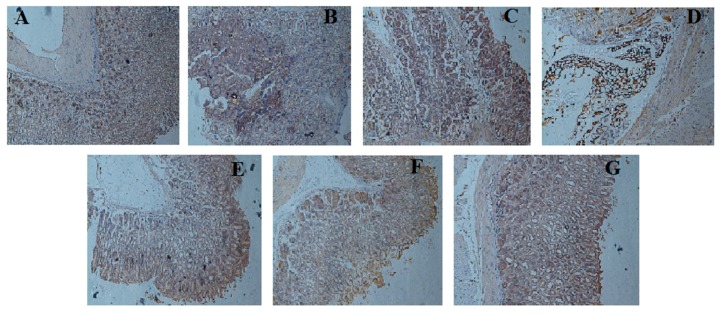
Immunohistochemical staining for the determination of NF-κBp65 protein expression in gastric mucosa. (**A**) Control; (**B**) model; (**C**) ethanol + OME 20 mg/kg; (**D**) ethanol + LCA 50 mg/kg; (**E**) ethanol + LCA 100 mg/kg; (**F**) ethanol + LCA 200 mg/kg; (**G**) ethanol + LCA 400 mg/kg. (Original magnification 100×).

Our results indicated that the expression of NF-κBp65 in the ethanol administered model group was clearly increased when compared to the control group (*f <* 0.01). These expressions was observed to be elevated epithelium and inflammatory infiltrate cell surfaces of the model group. LCA pretreatment significantly reduced the elevated expression of NF-κBp65 protein in a concentration dependent manner (Figure 7).

## 3. Discussion

There is increasing evidence that suggests the use of medicinal plants as an alternative option for the treatment of several gastrointestinal diseases including gastric ulcer. This has led to a rapid interest in the identification of bioactive natural products with gastroprotective properties which could be possible replacement for the synthetic available drugs with undesirable side effects. We have investigated the gastroprotective activity of the aerial parts of *Lycium chinense* Mill (LCA) in ethanol-induced gastric injury using mice models. The administration of ethanol (0.2 mL) produced acute inflammatory response, however pretreatment with various concentration of LCA effectively caused a reduction in the gastric injury induced by ethanol.

The infiltration and accumulation of leucocytes in the gastric mucosa tissues was evaluated using MPO activity. This assay has been widely used as a standard index for assessing the level of neutrophil infiltration in several experimental gastric injuries, colitis and human gastric ulcer [16]. It is widely believed that the ingestion of ethanol could induce acute inflammatory responses which lead to severe gastric injury. Our study indicated a marked increase in the activity of MPO in the mice stomach after the administration of ethanol, however, pretreatment with LCA prior to ethanol induced gastric ulcer significantly decreased the levels of MPO activity in the stomach. The affirmation of the protective effects of LCA was achieved by comparing the gastric injuries inflicted by the administration of ethanol in the model mice group to the stomachs of the mice in the normal control group as well as the mice groups treated with LCA. The protective effect of LCA was observed to be significant and in a concentration dependent manner. The prevention of the infiltration and accumulation of leucocytes, reduced edema of submucosal layer and the alleviation of necrotic lesions in the mice group pretreated with LCA for 7 days confirms the protective effects of LCA.

Adequate and quick inflammatory response is an integral and essential defense mechanism of the mucosa membrane [17]. The pro-inflammatory cytokines TNF-α has been implicated in inflammation, injuries and damages of various tissues and is secreted at the onset of gastric ulcer [18,19]. It is also actively involved in the stimulation and subsequent accumulation of neutrophil in the gastric mucosa [20]. IL-6 is released by T cells and macrophages as an immediate immune response during infection and tissue damages which leads to inflammation, as such they play an important role in acute inflammation and immune regulation [21]. The oxidative pathways which causes tissue damages during ulcer results from lymphocytes and neutrophils which are stimulated by the increased levels of IL-6. It has been proposed that TNF-α and IL-6 are critical in controlling the graveness of gastric ulcer and the release of these cytokines can lead to the accumulation of ROS in the mitochondria resulting to oxidative stress [22,23]. The results from this study indicated that LCA could prevent the elevated levels of these pro-inflammatory cytokines observed in the ethanol-induced gastric ulcer models, indicating its anti-inflammatory properties.

The consumption of ethanol elevates the level of MDA, a byproduct of lipid peroxidation and subsequently decreases the levels of antioxidant defense enzymes (SOD and GSH) as well as other factors involved in the protection of the gastric mucosa [24]. GSH is responsible for protecting tissues in the gastric mucosa from damages inflicted by free radical species, while SOD rapidly converts peroxyl radicals into biologically safe inactive substances [25,26]. The oral administration of LCA prior to ethanol-induced ulcer prevented the increased levels of MDA and elevated GSH and SOD levels in the treated mice models when compared with the untreated models, thereby attenuating the effects of ROS and oxidative stress caused by ethanol administration. This suggest that LCA possesses significant antioxidant properties.

NF-κB is a protein complex that controls transcription of DNA, production of cytokine and survival of cell. It is also involved in controlling pro-inflammatory mediators. The activation of this protein results in the production of inflammatory cytokines notably TNF-α, IL-1β and IL-6, which can aid the pathogenesis of inflammatory disorders [27]. ROS and TNF-α that are liberated in inflammatory processes could trigger NF-κB, which in turn activates inflammatory reactions. Furthermore, TNF-α can cause the degradation of IκB kinase whose function is to inactivate NF-κB by shielding the nuclear localization signals of NF-κB proteins, thus keeping it in its inactive form in the cytoplasm [28]. However, upon its activation, NF-κB moves to the nucleus and subsequently activates the consensus sequence related gene, such as TNF-α, IL-6, IL-2, IL-8, ICAM-1 which are involved in immune and inflammatory responses [29,30]. Immunohistochemical analysis indicated that the expression of NF-κB p65 was markedly increased in mice that were induced with ulcer as a result ethanol administration. This was however observed to be decreased in mice groups that received pretreatment with LCA for 7 days in a dose dependent manner. This result suggests that the protective effect of LCA might be partly due to the suppression of the activation of NF-κB pathway.

Previous report have isolated polysaccharides from *Lycium barbarum* a closely related specie of *Lycium chinense* and found that the polysaccharides isolated displayed anti-ulcer properties against water-immersion restraint stress, acetic acid and pylorus-ligation in rats [31]. The potential bioactivities of plants in this genus especially *Lycium chinense* is attributable to its vast chemical diversity and flavonoids are believed to play a pivotal role in these bioactivities as indicated in our study.

## 4. Experimental Section

### 4.1. Chemical and Reagent

Omeprazole (OME) was obtained from Shanghai XinyiJiahua Pharmaceutical Company Limited (Shannxi, China). MDA, GSH, SOD, and MPO kits were procured from the Nanjing Jiancheng Bioengineering Institute (Nanjing, China). The enzyme-linked immunosorbent assay (ELISA) kits for determination of IL-6, IL-1β and TNF-α were obtained from Nanjing KeyGEN Biotech. Co. Ltd. (Nanjing, China). NF-κBp65 polyclonal antibody was purchased from Santa Cruz Biotechnology Inc. (Santa Cruz, CA, USA). All other chemicals and reagents used were of analytical grade.

### 4.2. Plant Material

*Lycium chinense* Mill was collected at the medicinal plant garden of Jiangsu University, Zhenjiang, China. It was authenticated by one of the authors (Hongxia Chen) and a voucher specimen (LC 2015/101) was deposited at the herbarium of the university.

#### Extraction of Plant Material

The aerial part of *Lycium chinense* Mill (10 kg) was dried, ground and extracted with 90% EtOH (3 × 10 L) under reflux. The solution obtained was concentrated under reduced pressure, re-suspended in H_2_O and then successively extracted with hexane, EtOAc and *n*-BuOH. The EtOAc fraction (78 g) was fractionated using silica gel column chromatography eluting with CH_2_Cl_2_/CH_3_OH (1:0, 9:1, 8:2, 7:3, 6:4, 1:1, 0:1) to afford nine fractions. Fractions 2–4 was combined (5 g) and separated using silica gel column eluting with petroleum ether/EtOAc (10:1 to 1:1) to obtain four major fractions B2a–B2d. B2b was purified by semi-preparative HPLC usingCH_3_OH/H_2_O (75:25) to affords **1** (15 mg), **2** (10 mg) and **3** (22 mg). Fractions B23 was purified using silica gel column (petroleum ether/EtOAc (10:1 to 0:1) and semi-preparative HPLC using CH_3_OH/H_2_O (70:30) to obtain **4** (16 mg), **5** (10 mg) and **6** (4 mg).

### 4.3. Animals

Fifty six male ICR mice (25–30 g) were purchased from the experimental Animal Center of Jiangsu University (Zhenjiang, China). The animals were maintained in a temperature controlled room at 20.1–23.1 °C and 40%–50% humidity, a 12 h light/dark cycle and free access to food and water.

#### 4.3.1. Induction of Gastric Ulcer and Treatment

Mice were assigned into seven groups of eight mice per group: groups 1 (control) and 2 (model) were given distilled water orally. Group 3 was administered with omeprazole (OME) 20 mg/kg, while groups 4, 5, 6 and 7 were given LCA (50, 100, 200 and 400 mg/kg respectively). All drugs were administered once daily for 7 days. Drugs were given by gastric probe and were suspended in saline. On the last day of treatment, 90 mins after drug administration, the mice in groups 2–7 received absolute ethanol (0.2 mL/animal) orally, while group 1 received distilled water. After 4 h, mice were anaesthetized with ether and blood was collected through retro orbital puncture for biochemical estimation. The animals were sacrificed by cervical dislocation, the stomach was removed, opened along the greater curvature and rinsed gently in PBS. The serum and stomachs were subjected to further studies.

#### 4.3.2. Measurement of Gastric Juice Acid Content (pH)

The gastric contents in the stomach of each mouse was collected, centrifuge at 4000 rpm for 10 min. The pH of the supernatant for each sample was measured using a pH meter.

#### 4.3.3. MPO, MDA, GSH and SOD Levels Determination

MPO activity in stomachs, MDA, GSH, and SOD levels in serum were determined using commercially available kits obtained from Nanjing Jiancheng Bioengineering Institute (Nanjing, China) according to the manufacturer’s instructions.

#### 4.3.4. Cytokines Determination

The levels of serum IL-6, TNF-α and IL-1β were measured by ELISA according to the manufacturer’s instructions.

#### 4.3.5. Histological Procedure and Evaluation

The glandular stomach was fixed in 10% formalin solution for 24 h, sectioned and embedded in paraffin. A 5 mm thick section of the stomach was deparaffinized, stained with hematoxylin-eosin and was examined under a light microscope. Specimens were further evaluated based on previously described specification [32]. In brief, a 1 cm portion of each histological section was analyzed for epithelial cell loss (score: 0–3), edema in the upper mucosa (score: 0–4), hemorrhagic damage (score: 0–4), and the presence of inflammatory cells (score: 0–3), giving a total score of 14. Thereafter, the evaluation of the sections were performed by a pathologist who has no prior information as regards the treatment.

#### 4.3.6. Immunohistochemical Analysis

For the immunohistochemical evaluation, 5 mm thick sections of the stomach from each experimental animal group were prepared and immunohistochemical analysis was performed according to the previously described protocol [33]. Briefly, the sections were deparaffined, rehydrated, and pre-treated using citrate buffer (pH 6.0). Endogenous peroxide was blocked using 3% H_2_O_2_ for 15 min. The sections were further washed with PBS and blocked with 10% rabbit serum for 20 min to decrease the non-specific antibody-binding and then incubated with primary antibody (nuclear factor-κB p65, ratio of 1:100) at 4 °C overnight. The sections were rinsed with PBS and reacted with secondary antibodies and a polymer helper for 20 min at 37 °C and poly-HRP anti-goat IgG for 20 min at 37 °C. Slides were rewashed with PBS and developed using 3,3′-diaminobenzidine (DAB) and counterstained with hematoxylin.

### 4.4. Statistical Analysis

Statistical analysis was performed using SPSS (version 16.0, SPSS Inc., Chicago, IL, USA). All data were expressed as mean ± SD. Statistical analysis was performed one way Analysis of Variance (ANOVA) followed by Post hoc analysis using Tukey test. Differences were considered significant at *p* < 0.05.

## 5. Conclusions

In conclusion, the results obtained demonstrated that pretreatment with the aerial parts of *Lycium chinense* (LCA) exerts gastroprotective properties against ethanol-induced gastric ulcer in mice models. The underlying mechanisms involved in the anti-ulcerogenic effects might be related to the scavenging of oxidative free radicals, up regulation of antioxidant and anti-inflammatory status, reduced expression of pro-inflammatory mediators and the reduced expression of NF-κB pathway. LCA might have the potential for further development as a promising alternative for ulcer treatment. Further research to investigate the underlying molecular mechanism involved in the upstream pathways of TNF-α and NF-κBp65 pathways and the elucidation of the bioactive principles are in process.
